# 4-(4-Pyridylamino)pyridinium perchlorate

**DOI:** 10.1107/S1600536809003353

**Published:** 2009-01-31

**Authors:** Gregory A. Farnum, Robert L. LaDuca

**Affiliations:** a Lyman Briggs College, Department of Chemistry, Michigan State University, East Lansing, MI 48825, USA

## Abstract

In the title salt, C_10_H_10_N_3_^+^·ClO_4_^−^, the 4-(4-pyridylamino)­pyridinium cations are linked into chains *via* N—H⋯N hydrogen bonding and into layers by C—H⋯π inter­actions [C⋯*Cg* = 3.3875 (19) Å]. Perchlorate ions are anchored to the layer motifs by N—H⋯O hydrogen bonding. The perchlorate anion was found to be disordered about a Cl—O axis, with two sites, each of equal occupancy, being resolved for the three remaining O atoms.

## Related literature

For divalent metal adipate coordination polymers incorporating 4,4′-dipyridylamine as a ligand, see: Montney *et al.* (2007 ▶).
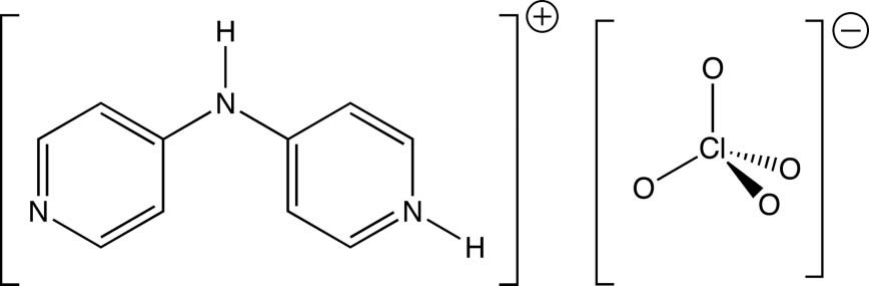


## Experimental

### 

#### Crystal data

C_10_H_10_N_3_^+^·ClO_4_^−^
*M* *_r_* = 271.66
Monoclinic, 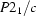

*a* = 7.6254 (10) Å
*b* = 15.991 (2) Å
*c* = 9.8358 (13) Å
β = 101.913 (1)°
*V* = 1173.5 (3) Å^3^
*Z* = 4
Mo *K*α radiation
μ = 0.34 mm^−1^
*T* = 173 (2) K
0.36 × 0.24 × 0.18 mm

#### Data collection

Bruker SMART 1K diffractometer
Absorption correction: multi-scan (*SADABS*; Sheldrick, 2007 ▶) *T*_min_ = 0.907, *T*_max_ = 0.941
12615 measured reflections
2728 independent reflections
2165 reflections with *I* > 2σ(*I*)
*R*_int_ = 0.039

#### Refinement

*R*[*F* ^2^ > 2σ(*F* ^2^)] = 0.037
*wR*(*F* ^2^) = 0.096
*S* = 1.03
2728 reflections
196 parameters
H atoms treated by a mixture of independent and constrained refinement
Δρ_max_ = 0.39 e Å^−3^
Δρ_min_ = −0.32 e Å^−3^

### 

Data collection: *SMART* (Bruker, 2006 ▶); cell refinement: *SAINT* (Bruker, 2006 ▶); data reduction: *SAINT*; program(s) used to solve structure: *SHELXS97* (Sheldrick, 2008 ▶); program(s) used to refine structure: *SHELXL97* (Sheldrick, 2008 ▶); molecular graphics: *CrystalMaker* (Palmer, 2007 ▶); software used to prepare material for publication: *SHELXL97*.

## Supplementary Material

Crystal structure: contains datablocks I, global. DOI: 10.1107/S1600536809003353/tk2362sup1.cif

Structure factors: contains datablocks I. DOI: 10.1107/S1600536809003353/tk2362Isup2.hkl

Additional supplementary materials:  crystallographic information; 3D view; checkCIF report

## Figures and Tables

**Table 1:** Hydrogen-bond geometry (Å, °)

*D*—H⋯*A*	*D*—H	H⋯*A*	*D*⋯*A*	*D*—H⋯*A*
N1—H1N⋯N3^i^	0.85 (2)	2.04 (2)	2.839 (2)	157 (2)
N2—H2N⋯O3*A*^ii^	0.85 (2)	2.17 (2)	2.873 (8)	140.2 (18)
N2—H2N⋯O4^ii^	0.85 (2)	2.27 (2)	3.098 (5)	166.1 (19)

Symmetry codes: (i) 
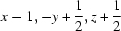
; (ii) 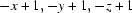.

**Fig. 1.**
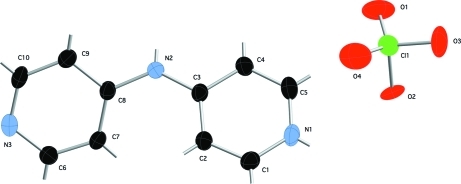
The asymmetric unit of (I), showing 50% probability ellipsoids and atom numbering scheme. H atom positions are shown as grey sticks. Only one of the disordered set of perchlorate positions is shown. Colour code: green Cl, light blue N, red O, black C.

**Fig. 2.**
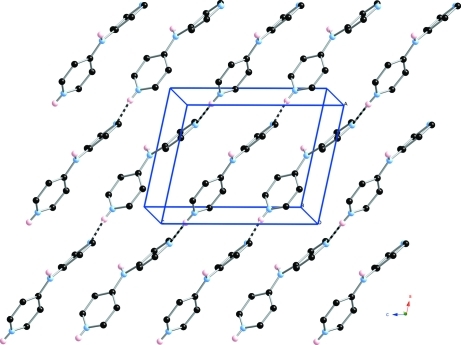
A layer of [C10H10N3]+ cations in (I). The N—H···N interactions are depicted as dashed lines.

**Fig. 3.**
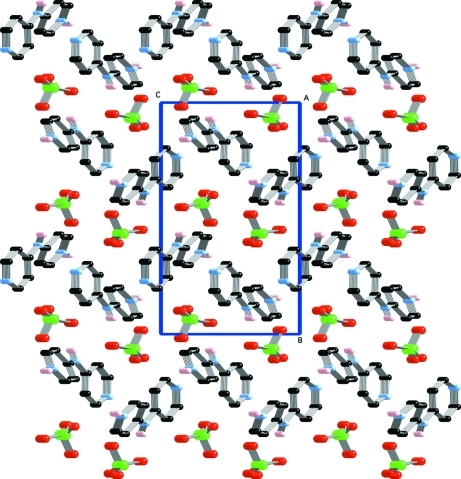
Stacking diagram for (I), viewed slightly offset from the b-direction.

## supplementary crystallographic information

### Comment

The dipodal tethering ligand 4,4'-dipyridylamine (dpa) has proven beneficial for the construction of divalent metal adipate coordination polymers with novel topologies (Montney et al., 2007). In an attempt to probe the effect of alkyl group substitution on coordination polymer structure by using methyladipate, colourless crystals of the title salt (I) were obtained.

The asymmetric unit of (I) comprises a Hdpa^+^ cation and a perchlorate ion, with three of its O atoms disordered equally over two positions (Fig. 1). The Hdpa^+^ cations aggregate into supramolecular chains, aligned along [201] by means of N—H···N hydrogen bonding interactions between protonated and unprotonated pyridyl rings, Table 1. These chains are organized into layers (Fig. 2), oriented parallel to the ac-plane, and connected by C—H···π interactions between pyridyl rings in neighbouring Hdpa^+^ cations [C1—H1···Cg(N2,C6–C10)^i^ = 2.82 Å, C1···Cg^i^ = 3.3875 (19) Å with angle at H1 = 119° for i = -1+x, y, z]. Supramolecular interactions are optimized by the 33.77 (8)° torsion angle between the pyridyl rings. Perchlorate anions are anchored to the layer motifs by N—H···O hydrogen bonding; the layers stack along the b-direction (Fig. 3)

### Experimental

All chemicals were obtained commercially. Cadmium perchlorate hydrate (20 mg, 0.064 mmol) and methyladipic acid (10 mg, 0.064 mmol) were dissolved in water (1.5 ml) in a glass vial. A 0.75 ml aliquot of a 1:1 water:ethanol mixture was carefully layered onto the aqueous solution, followed by an ethanolic solution (1.5 ml) of 4,4'-dipyridylamine (22 mg, 0.12 mmol). Colourless blocks of the title salt formed after 2 weeks.

### Refinement

All H atoms bound to C atoms were placed in calculated positions, with C—H = 0.95 Å and refined in riding mode with *U*_iso_ = 1.2*U*_eq_(C). The H atoms bound to N atoms were found *via* a Fourier difference map, restrained with N—H = 0.85 (2) Å, and refined with *U*_iso_ = 1.2*U*_eq_(N). The perchlorate was disordered about a Cl—O axis with two sites, each of equal occupancy, being resolved for the three remaining O atoms. All atoms of the disordered model were refined anisotropcially.

### Crystal data

**Table:**

C_10_H_10_N_3_^+^·ClO_4_^−^	*F*(000) = 560
*M**_r_* = 271.66	*D*_x_ = 1.538 Mg m^−^^3^
Monoclinic, *P*2_1_/*c*	Mo *K*α radiation, λ = 0.71073 Å
Hall symbol: -P 2ybc	Cell parameters from 12615 reflections
*a* = 7.6254 (10) Å	θ = 2.5–28.3°
*b* = 15.991 (2) Å	µ = 0.34 mm^−^^1^
*c* = 9.8358 (13) Å	*T* = 173 K
β = 101.913 (1)°	Block, colourless
*V* = 1173.5 (3) Å^3^	0.36 × 0.24 × 0.18 mm
*Z* = 4	

### Data collection

**Table:**

Bruker SMART 1K diffractometer	2728 independent reflections
Radiation source: fine-focus sealed tube	2165 reflections with *I* > 2σ(*I*)
graphite	*R*_int_ = 0.039
ω scans	θ_max_ = 28.3°, θ_min_ = 2.5°
Absorption correction: multi-scan (*SADABS*; Sheldrick, 2007)	*h* = −9→9
*T*_min_ = 0.907, *T*_max_ = 0.941	*k* = −20→20
12615 measured reflections	*l* = −12→12

### Refinement

**Table:**

Refinement on *F*^2^	Primary atom site location: structure-invariant direct methods
Least-squares matrix: full	Secondary atom site location: difference Fourier map
*R*[*F*^2^ > 2σ(*F*^2^)] = 0.037	Hydrogen site location: inferred from neighbouring sites
*wR*(*F*^2^) = 0.096	H atoms treated by a mixture of independent and constrained refinement
*S* = 1.03	*w* = 1/[σ^2^(*F*_o_^2^) + (0.0377*P*)^2^ + 0.7438*P*] where *P* = (*F*_o_^2^ + 2*F*_c_^2^)/3
2728 reflections	(Δ/σ)_max_ < 0.001
196 parameters	Δρ_max_ = 0.39 e Å^−^^3^
0 restraints	Δρ_min_ = −0.32 e Å^−^^3^

### Special details

**Table:**

Geometry. All e.s.d.'s (except the e.s.d. in the dihedral angle between two l.s. planes) are estimated using the full covariance matrix. The cell e.s.d.'s are taken into account individually in the estimation of e.s.d.'s in distances, angles and torsion angles; correlations between e.s.d.'s in cell parameters are only used when they are defined by crystal symmetry. An approximate (isotropic) treatment of cell e.s.d.'s is used for estimating e.s.d.'s involving l.s. planes.
Refinement. Refinement of *F*^2^ against ALL reflections. The weighted *R*-factor *wR* and goodness of fit *S* are based on *F*^2^, conventional *R*-factors *R* are based on *F*, with *F* set to zero for negative *F*^2^. The threshold expression of *F*^2^ > σ(*F*^2^) is used only for calculating *R*-factors(gt) *etc*. and is not relevant to the choice of reflections for refinement. *R*-factors based on *F*^2^ are statistically about twice as large as those based on *F*, and *R*- factors based on ALL data will be even larger.

### Fractional atomic coordinates and isotropic or equivalent isotropic displacement parameters (Å^2^)

**Table:**

	*x*	*y*	*z*	*U*_iso_*/*U*_eq_	Occ. (<1)
Cl1	0.21340 (6)	0.43473 (3)	0.70621 (5)	0.03353 (14)	
O2	0.2109 (9)	0.5174 (3)	0.6429 (5)	0.0405 (12)	0.50
O3	0.3562 (15)	0.4011 (7)	0.6502 (10)	0.057 (2)	0.50
O4	0.2567 (7)	0.4511 (3)	0.8496 (4)	0.0676 (13)	0.50
O2A	0.2135 (9)	0.4032 (7)	0.8406 (6)	0.169 (4)	0.50
O3A	0.2109 (10)	0.5200 (5)	0.6966 (10)	0.124 (4)	0.50
O4A	0.3645 (17)	0.3918 (7)	0.6858 (13)	0.095 (4)	0.50
O1	0.0503 (2)	0.39806 (10)	0.6382 (2)	0.0597 (5)	
N1	0.0749 (2)	0.35317 (10)	0.28489 (16)	0.0309 (3)	
H1N	−0.011 (3)	0.3422 (13)	0.325 (2)	0.037*	
N2	0.52241 (19)	0.38493 (9)	0.11764 (16)	0.0280 (3)	
H2N	0.579 (3)	0.4301 (13)	0.141 (2)	0.034*	
N3	0.8017 (2)	0.22960 (10)	−0.10972 (16)	0.0318 (3)	
C1	0.1057 (2)	0.29873 (11)	0.18885 (19)	0.0309 (4)	
H1	0.0253	0.2534	0.1628	0.037*	
C2	0.2499 (2)	0.30704 (11)	0.12757 (19)	0.0287 (4)	
H2	0.2679	0.2687	0.0579	0.034*	
C3	0.3713 (2)	0.37270 (10)	0.16845 (17)	0.0245 (3)	
C4	0.3305 (2)	0.43069 (11)	0.26600 (18)	0.0290 (4)	
H4	0.4060	0.4777	0.2926	0.035*	
C5	0.1831 (2)	0.41920 (12)	0.32180 (19)	0.0321 (4)	
H5	0.1565	0.4583	0.3875	0.039*	
C6	0.6948 (2)	0.19706 (12)	−0.03119 (19)	0.0296 (4)	
H6	0.6856	0.1379	−0.0275	0.036*	
C7	0.7192 (2)	0.36562 (11)	−0.04063 (19)	0.0303 (4)	
H7	0.7312	0.4246	−0.0461	0.036*	
C8	0.6077 (2)	0.33074 (10)	0.04003 (17)	0.0247 (3)	
C9	0.5971 (2)	0.24396 (11)	0.04488 (18)	0.0272 (4)	
H9	0.5240	0.2175	0.0995	0.033*	
C10	0.8118 (2)	0.31334 (12)	−0.11227 (19)	0.0335 (4)	
H10	0.8873	0.3381	−0.1667	0.040*	

### Atomic displacement parameters (Å^2^)

**Table:**

	*U*^11^	*U*^22^	*U*^33^	*U*^12^	*U*^13^	*U*^23^
Cl1	0.0299 (2)	0.0328 (2)	0.0376 (3)	0.00006 (18)	0.00622 (17)	−0.00398 (18)
O2	0.049 (2)	0.022 (2)	0.055 (2)	−0.0091 (17)	0.0214 (18)	0.0102 (17)
O3	0.044 (4)	0.071 (5)	0.065 (3)	0.011 (3)	0.034 (3)	−0.009 (2)
O4	0.107 (4)	0.063 (3)	0.0306 (18)	−0.023 (2)	0.010 (2)	−0.0062 (17)
O2A	0.115 (5)	0.327 (12)	0.050 (3)	−0.121 (7)	−0.018 (3)	0.064 (5)
O3A	0.037 (3)	0.037 (3)	0.279 (11)	0.005 (2)	−0.006 (5)	−0.067 (5)
O4A	0.047 (4)	0.045 (3)	0.183 (11)	0.022 (3)	−0.002 (5)	−0.033 (6)
O1	0.0370 (8)	0.0382 (8)	0.0990 (14)	−0.0130 (7)	0.0028 (8)	−0.0019 (9)
N1	0.0262 (7)	0.0354 (8)	0.0359 (8)	0.0032 (6)	0.0175 (6)	0.0061 (7)
N2	0.0236 (7)	0.0258 (7)	0.0379 (8)	−0.0042 (6)	0.0140 (6)	−0.0066 (6)
N3	0.0298 (8)	0.0373 (9)	0.0313 (8)	0.0015 (6)	0.0133 (6)	−0.0035 (6)
C1	0.0249 (8)	0.0270 (9)	0.0427 (10)	0.0002 (7)	0.0112 (8)	0.0035 (8)
C2	0.0255 (8)	0.0281 (9)	0.0350 (9)	0.0008 (7)	0.0115 (7)	−0.0041 (7)
C3	0.0222 (8)	0.0256 (8)	0.0271 (8)	0.0029 (6)	0.0084 (6)	0.0030 (7)
C4	0.0269 (8)	0.0273 (9)	0.0339 (9)	0.0007 (7)	0.0091 (7)	−0.0042 (7)
C5	0.0304 (9)	0.0366 (10)	0.0316 (9)	0.0079 (8)	0.0114 (7)	−0.0014 (8)
C6	0.0256 (8)	0.0289 (9)	0.0357 (9)	−0.0009 (7)	0.0095 (7)	−0.0036 (7)
C7	0.0286 (9)	0.0290 (9)	0.0360 (9)	−0.0008 (7)	0.0129 (7)	0.0030 (7)
C8	0.0199 (7)	0.0298 (9)	0.0252 (8)	0.0001 (6)	0.0067 (6)	−0.0025 (7)
C9	0.0227 (8)	0.0293 (9)	0.0323 (9)	−0.0021 (7)	0.0118 (7)	0.0007 (7)
C10	0.0311 (9)	0.0410 (11)	0.0331 (9)	−0.0016 (8)	0.0175 (8)	0.0015 (8)

### Geometric parameters (Å, °)

**Table:**

Cl1—O3A	1.366 (7)	C1—H1	0.9500
Cl1—O4A	1.391 (11)	C2—C3	1.402 (2)
Cl1—O4	1.405 (4)	C2—H2	0.9500
Cl1—O1	1.4125 (15)	C3—C4	1.414 (2)
Cl1—O2A	1.414 (5)	C4—C5	1.362 (2)
Cl1—O3	1.423 (8)	C4—H4	0.9500
Cl1—O2	1.459 (5)	C5—H5	0.9500
N1—C1	1.341 (2)	C6—C9	1.381 (2)
N1—C5	1.343 (2)	C6—H6	0.9500
N1—H1N	0.85 (2)	C7—C10	1.378 (2)
N2—C3	1.362 (2)	C7—C8	1.394 (2)
N2—C8	1.400 (2)	C7—H7	0.9500
N2—H2N	0.85 (2)	C8—C9	1.391 (2)
N3—C6	1.338 (2)	C9—H9	0.9500
N3—C10	1.342 (2)	C10—H10	0.9500
C1—C2	1.366 (2)		
			
O3A—Cl1—O4A	118.9 (6)	N2—C3—C2	124.10 (15)
O3A—Cl1—O1	112.5 (3)	N2—C3—C4	118.42 (16)
O4A—Cl1—O1	113.7 (5)	C2—C3—C4	117.46 (15)
O4—Cl1—O1	123.6 (2)	C5—C4—C3	120.02 (17)
O3A—Cl1—O2A	114.7 (6)	C5—C4—H4	120.0
O4A—Cl1—O2A	96.8 (7)	C3—C4—H4	120.0
O1—Cl1—O2A	97.2 (2)	N1—C5—C4	120.63 (17)
O4—Cl1—O3	114.8 (5)	N1—C5—H5	119.7
O1—Cl1—O3	109.2 (5)	C4—C5—H5	119.7
O4—Cl1—O2	103.9 (3)	N3—C6—C9	124.21 (17)
O1—Cl1—O2	103.9 (3)	N3—C6—H6	117.9
O3—Cl1—O2	96.9 (5)	C9—C6—H6	117.9
C1—N1—C5	120.91 (15)	C10—C7—C8	119.04 (16)
C1—N1—H1N	117.0 (14)	C10—C7—H7	120.5
C5—N1—H1N	121.9 (14)	C8—C7—H7	120.5
C3—N2—C8	129.38 (15)	C9—C8—C7	117.72 (15)
C3—N2—H2N	116.3 (14)	C9—C8—N2	124.18 (15)
C8—N2—H2N	114.1 (14)	C7—C8—N2	117.97 (15)
C6—N3—C10	116.29 (15)	C6—C9—C8	118.78 (15)
N1—C1—C2	121.48 (17)	C6—C9—H9	120.6
N1—C1—H1	119.3	C8—C9—H9	120.6
C2—C1—H1	119.3	N3—C10—C7	123.95 (16)
C1—C2—C3	119.35 (16)	N3—C10—H10	118.0
C1—C2—H2	120.3	C7—C10—H10	118.0
C3—C2—H2	120.3		
			
C5—N1—C1—C2	1.6 (3)	C10—N3—C6—C9	0.1 (3)
N1—C1—C2—C3	1.7 (3)	C10—C7—C8—C9	0.6 (3)
C8—N2—C3—C2	−13.5 (3)	C10—C7—C8—N2	176.70 (16)
C8—N2—C3—C4	168.15 (17)	C3—N2—C8—C9	−26.6 (3)
C1—C2—C3—N2	177.63 (17)	C3—N2—C8—C7	157.57 (18)
C1—C2—C3—C4	−4.0 (3)	N3—C6—C9—C8	0.5 (3)
N2—C3—C4—C5	−178.24 (16)	C7—C8—C9—C6	−0.8 (3)
C2—C3—C4—C5	3.3 (3)	N2—C8—C9—C6	−176.68 (16)
C1—N1—C5—C4	−2.4 (3)	C6—N3—C10—C7	−0.4 (3)
C3—C4—C5—N1	−0.2 (3)	C8—C7—C10—N3	0.0 (3)

### Hydrogen-bond geometry (Å, °)

**Table:**

*D*—H···*A*	*D*—H	H···*A*	*D*···*A*	*D*—H···*A*
N1—H1N···N3^i^	0.85 (2)	2.04 (2)	2.839 (2)	157 (2)
N2—H2N···O3A^ii^	0.85 (2)	2.17 (2)	2.873 (8)	140.2 (18)
N2—H2N···O4^ii^	0.85 (2)	2.27 (2)	3.098 (5)	166.1 (19)

Symmetry codes: (i) *x*−1, −*y*+1/2, *z*+1/2; (ii) −*x*+1, −*y*+1, −*z*+1.
